# Systematic calibration of a cell signaling network model

**DOI:** 10.1186/1471-2105-11-202

**Published:** 2010-04-23

**Authors:** Kyoung Ae Kim, Sabrina L Spencer, John G Albeck, John M Burke, Peter K Sorger, Suzanne Gaudet, Do Hyun Kim

**Affiliations:** 1 Department of Chemical and Biomolecular Engineering(BK21 Program) and Center for Ultramicrochemical Process Systems, Korea Advanced Institute of Science and Technology, 335 Gwahak-ro, Yuseong-gu, Daejeon, 305-701, Republic of Korea; 2 Center for Cell Decision Process, Department of Biological Engineering, Massachusetts Institute of Technology, Cambridge, Massachusetts, 02139, USA; 3 Department of Systems Biology, Harvard Medical School, Boston, Massachusetts, 02115, USA

## Abstract

**Background:**

Mathematical modeling is being applied to increasingly complex biological systems and datasets; however, the process of analyzing and calibrating against experimental data is often challenging and a rate limiting step in model development. To address this problem, we developed a systematic methodology for calibrating quantitative models of dynamic biological processes and illustrate its utility by validating a model of TRAIL (Tumor necrosis factor Related Apoptosis-Inducing Ligand)-induced cell death.

**Results:**

We propose a serial framework integrating analysis and calibration modules and we compare various methods for global sensitivity analysis and global parameter estimation. First, adequacy of the network structure is checked by global sensitivity analysis to changes in concentrations of molecular species, validating that the model can reproduce qualitative features of the system behavior derived from experiments or literature surveys. Second, rate parameters are ranked by importance using gradient-based and variance-based sensitivity indices, and we systematically determine the optimal number of parameters to include in model calibration. Third, deterministic, stochastic and hybrid algorithms for global optimization are applied to estimate the values of the most important parameters by fitting to time series data. We compare the performance of these three optimization algorithms.

**Conclusions:**

Our proposed framework covers the entire process from validating a proto-model to establishing a realistic model for *in silico *experiments and thereby provides a generalized workflow for the construction of predictive models of complex network systems.

## Background

Comprehensive and predictive models of biological systems are expected to improve our ability to analyze complex systems, from molecular pathways to populations of organisms. Thus, there is much interest in sophisticated computational modeling techniques and high-throughput data generation [1]. One of the major difficulties in modeling cell signaling networks is the identification of the directionality and strength of relationship between molecular species in specific pathways. However, once this has been done, the knowledge can be formalized in mathematical models based on various computational methods. In particular, differential equations are widely used in biological modeling to describe dynamic processes in terms of rates of change [2-4]. The variables in these models represent the concentrations of molecular species and the directionality and strength of their relationships are encoded in the rate parameters governing their interactions. Following the construction of a mathematical representation, cycles of experimental validation and model improvement are essential for generating a predictive model, by ensuring that all required molecular species are adequately represented and that the parameter values are accurate. However, calibration of the mathematical model is not trivial because non-linearity and feedback/feedforward connections commonly found in cell signaling pathways make the analysis difficult [5,6]. Here, we develop a systematic methodology for validating quantitative models of biological processes and apply our methodology to an existing model of TRAIL-induced apoptosis [7].

### Systematic procedure of model calibration

Model calibration or regression by data fitting is necessary for computational modeling in any field of science or engineering. Systems biology faces the same challenge to construct experimentally validated models. However, formal tools for quantitative biological models have not been established yet and manual analysis is common in practice. In fact, manual fitting has the advantage that researchers may apply their experimental intuition or prior knowledge to the model relatively easily with minimal aid of mathematical or computational skills. However, the structural complexity of signaling pathways makes it difficult to fit the model heuristically based on intuition or simple analyses only. There are three dominant differences between manual fitting and systematic calibration: (1) As in Yang's work [8], manual fitting is attempted to estimate uncertain parameter values which cannot be decided directly by experimental measurement or literature. On the other hand, the systematic calibration in our study aims principally to estimate, among uncertain parameters, only the most important. We investigated the individual effect of parameters and focused on the dominant parameters to calibrate the model. (2) Manual fitting is carried out mainly by a trial-and-error process that does not guarantee optimal fit of the model. On the other hand, our systematic calibration method approaches the problem globally over the multi-dimensional domain of important uncertain parameters. Thus, it has higher probability of finding the optimal solution. (3) Manual fitting ends with what are, at the time, the best parameter values, while systematic calibration provides additional information, such as important subsets of pathways in a network or possible local optimum solutions.

**Figure 1:**
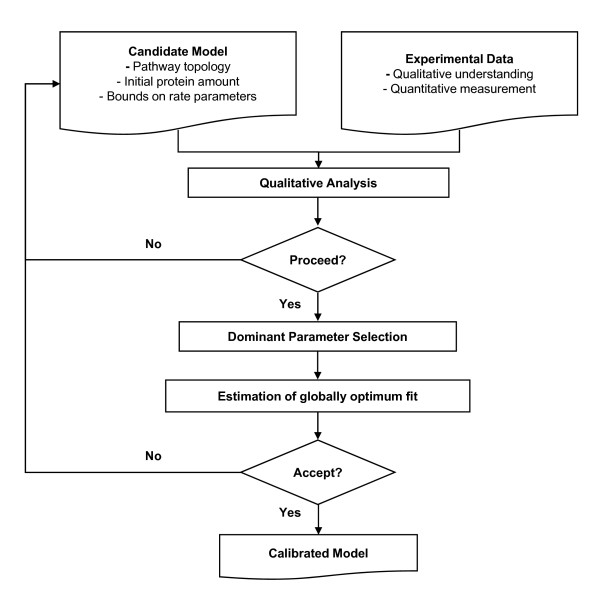
**Schematic workflow for efficient model calibration**.

We have developed a systematic calibration procedure for testing and improving models as shown in Figure 1. In the first step, the model is constructed based on information from the literature and analyzed qualitatively to ensure that it is in agreement with prior knowledge about the network. Usually, the construction of the network model is based on information from the literature and published experimental results are what we aim to qualitatively reproduce. Because only the structural characteristics of the model are of interest in this step, a model with tentative parameter values is not necessarily expected to reproduce experimental data quantitatively. The suitability of the proto-model can be assessed by analyzing output sensitivities to input variables values under presumed uncertainties of the rate parameters (Figure 1; Qualitative analysis). Candidate model modifications are iterated until a satisfactory qualitative match to the prior knowledge is obtained.

In the second step, we assess whether a subset of pathway reactions dominantly affects the model outputs, focusing on the outputs that are described by the experimental data to be used in the calibration step (Figure 1; Dominant Parameter Selection). Identification of dominant reactions is done by sensitivity analysis; many methods exist [9-14] and we adopted two methods that are most appropriate to nonlinear network models (Table 1).

**Table 1:** The computational methods used for analyzing the network model

	Computational methods	Searching principle	Reference
Sensitivity analysis	Local sensitivity	Local	
	
	Average of local sensitivities	Global	[17]
	
	Sobol's method		[11]
	First-order sensitivity	Global	
	Total-effect sensitivity	Global	

Parameter estimation	Local estimation	Local, deterministic	
	
	Multi-start of local estimations	Global, deterministic	
	
	Evolutionary strategy using stochastic ranking (SRES)	Global, stochastic	[22]

In the third step, we perform a quantitative fit, or calibration, of the model to experimental data, to determine parameter values that minimize the deviations between experimental results and model simulations (Figure 1; Estimation of globally optimal fit). Parameter estimation by global optimization has been developed for engineering optimization problems [15,16]. Below we investigate the advantages and disadvantages of three methods for biological applications, including computational efficiency, and compare the results (Table 1).

Lastly, as the model evolves in light of newly available data, the overall procedure should be iterated. We believe that by implementing the intermediary steps where sensitivity analyses are used both to assess the qualitative behavior of the model and determine which parameters to optimize, our systematic method will significantly facilitate model calibration.

## Results and discussion

### Qualitative analysis of a proto-model

Analysis of sensitivity with respect to initial species concentrations provides a criterion for the qualitative correctness of a cell signaling model. Sensitivity analysis assesses how changes in model inputs contribute to model output variability, and its ability to deduce model input-output relationships makes sensitivity analysis one of the critical parts of model development, verification, and evaluation. Changes in initial species concentrations can mimic the effects of mutations or changes in the expression level of the molecular players involved, and the sensitivity of the model output to changes in initial species concentrations should match the expected change in system behavior.

The simplest and most generally used sensitivity analysis method is a gradient-based index as follows,

where model outputs and inputs are represented as *y*_*i *_and *p*_*j *_respectively. This method is often called local sensitivity because it reflects output variability accurately near a given nominal input value, p*. However, most kinetic parameters are quite uncertain and a range rather than a single parameter value is available, either from the literature or from biophysical constraints on the reactions. Thus, "model-independent", or more precisely, parameter-independent, global sensitivity analysis techniques have generated great interest[10]. Averaging of local sensitivities over a range of plausible values for uncertain parameters is one possible method for global sensitivity. Local sensitivities are calculated with multiple parameter choices that are selected randomly or regularly within parameter ranges. The sensitivities for those parameter choices, integrated over the time interval of interest in the monitoring of the output, ∫|*S*_*ij*_(*t*)|*dt*, are then averaged to determine global sensitivity [17]. Importantly, because integration over time and averaging are necessary, a compromise must be made between accurately calculating the magnitude of the effects by using absolute sensitivity values, and assessing the directionality of the effects, by maintaining the sign of the values.

The model on which we applied our methodology simulates the response of a single cell to TRAIL. TRAIL is a protein ligand which triggers the process of programmed cell death, or apoptosis. This model of TRAIL-induced cell death signaling network encompasses the activation of initiator (caspase-8 or C8) and effector (caspase-3 or C3) caspases, the onset of mitochondrial outer membrane permeabilization and the death of the cell, as marked by cleavage of the caspase-3 substrate, PARP. According to a recent study [7], extrinsic apoptosis shows a specific behavior of all-or-none effector caspase activation at the single-cell level. As the authors termed it, the process shows "variable-delay, snap-action": a long, variable delay between TRAIL stimulation and effector caspase activation is followed by rapid and sudden progression to completion. The original model is composed of 58 ordinary differential equations based on mass action kinetics. Eighteen out of 58 protein species have non-zero initial concentrations, and 70 rate constants regulate the reactions in the model network. The original parameters were determined from the literature and manual fitting. In this study, we applied our methods to analyze qualitative properties of the model and fit the model to dynamic quantitative experimental data in a systematical and computationally effective way. Hereafter, the original model in [7] will be referred to as the manually calibrated model, to distinguish it from our improved model.

**Figure 2:**
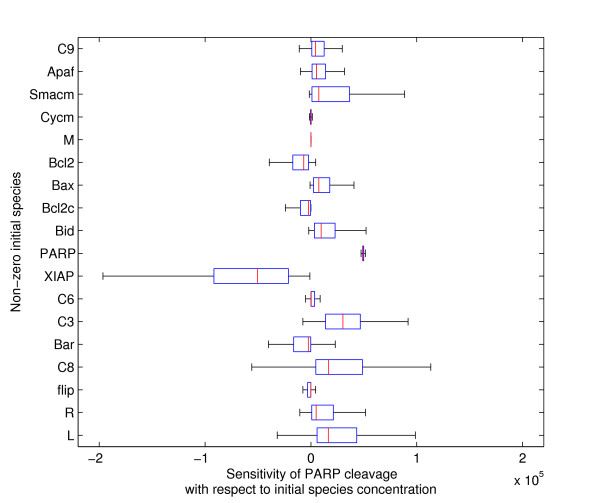
**Distribution of sensitivity of PARP cleavage**. Each box plot shows the distribution of sensitivity of PARP cleavage with respect to change in non-zero initial species concentrations determined by average of local gradient-based sensitivities.

In our analysis, cleavage of PARP is the key output; because the process is all-or-none, if >50% of PARP is cleaved, it is eventually all cleaved and thus a simulated cell is deemed dead at 50% cleaved PARP (see Methods). We first evaluated sensitivities of the cleavage of PARP with respect to changes in initial species concentrations, sampling over a range of plausible parameter values (range described in Methods). In this case, instead of averaging the sensitivities over the sampled range of parameter values, we plotted their distributions in a box plot, to preserve the directionality of the effect in the sign of the sensitivities (Figure 2). We interpreted the results in three ways. First, proteins with positive sensitivity would promote PARP cleavage and thus were pro-apoptotic, and by corollary, proteins with negative sensitivity would repress PARP cleavage and had an anti-apoptotic effect. The pro- or anti-apoptotic nature of TRAIL-induced signaling proteins has been identified in the literature and should be encoded properly in the mathematical model. For instance, XIAP (an inhibitor of caspase-3) is a well known anti-apoptotic player, and the sensitivity of PARP cleavage with respect to XIAP was correctly shown to be negative (Figure 2). Conversely, Smac, when released from the mitochondria, inhibits XIAP and thus the positive sign of sensitivity with respect to the mitochondrial store of Smac, Smac_m_, agrees with its pro-apoptotic nature (Figure 2). By similarly assessing the sign of the sensitivity of each protein, the TRAIL-induced cell death proto-model could be validated.

Second, the absolute value of sensitivity provides a measure of how strongly the perturbation of a single species' concentration affects the model output. The sensitivity with respect to perturbation of XIAP was found to be relatively high on average, implying that the model output can be changed dramatically by small changes in XIAP (Figure 2). This prediction is supported by biological evidence that XIAP directly inhibits the enzymatic activity of caspases and the degree of inhibition is highly dependent on the concentration of XIAP[18]. Cleavage of PARP is insensitive to the concentration of caspase-6 (C6), in agreement with experiments in which reducing the expression of caspase-6 by ~90% did not affect TRAIL-induced cell death (Figure 2; [7]). Overall, our sensitivity analysis agreed with the known effects of varying protein concentration. If, however, the signs or strengths of the sensitivities in our analysis had not agreed with experimental results, the model construction would have to be re-examined. Modification of the model and this qualitative analysis would be done iteratively until a satisfactory result could be reached. Although the TRAIL model study does not provide us with an example of failure of qualitative agreement at this step of the procedure, it is still worth noting that qualitative agreement with known experimental system behavior can be a strong preliminary criteria for adequacy of the model structure. In effect, it sets a minimal qualification that must be met before more computationally intensive methods are applied to improve the proto-model by quantitative fitting.

In a third type of assessment of the results our sensitivity analysis of PARP cleavage, we analyzed the influence of the uncertainty of rate constants on the sensitivity with respect to initial species concentrations. Sensitivities that are not affected by parameter values will have narrow distributions, and by consequence, their sensitivity value is very reliable. The sensitivities related to the perturbation of some species like XIAP and caspase-8 were found to be broadly distributed and thus to be relatively uncertain (Figure 2). Particularly interesting is the fact that the sensitivity of PARP cleavage to caspase-8 is negative in some cases, even if it is known to have a pro-apoptotic function, invalidating certain parameter sets.

### Dominant parameter selection

**Figure 3:**
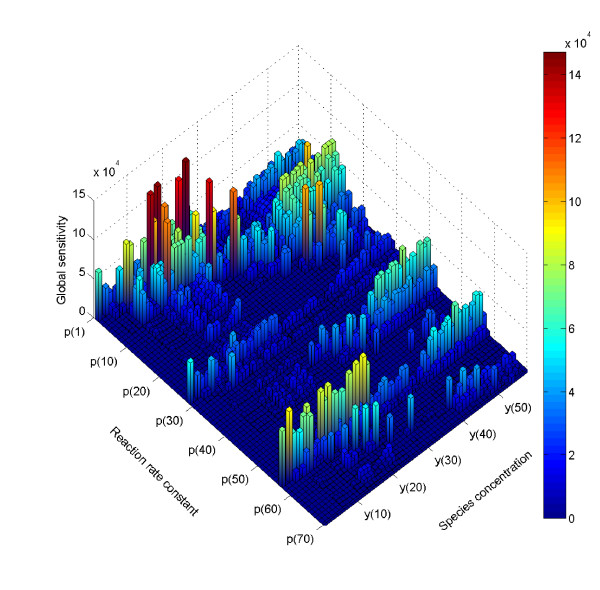
**Global sensitivity matrix of TRAIL-induced cell death model**. The height of bars represents the global sensitivity for all 58 model outputs with respect to change in 70 kinetic reaction rate constants, as determined by average of local gradient-based sensitivities.

**Figure 4:**
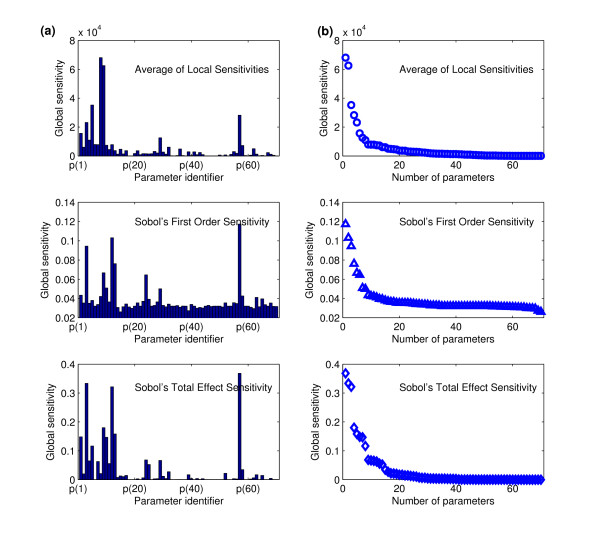
**Comparison of different global sensitivity algorithms**. Three different sensitivities, which are average of local sensitivities, Sobol's first order sensitivity, and Sobol's total effect sensitivity, are compared. (a) Bar graph showing the global sensitivity for PARP cleavage for the 70 kinetic parameters. (b) Plot showing the global sensitivity for PARP cleavage vs. parameter number, for parameters sorted according to the magnitude of their sensitivity

When global sensitivities are determined by averaging local sensitivities as we did above, no assumptions are made in the relationships between input parameters and output variables, so this method is applicable in most nonlinear and non-monotonic problems. For the model of TRAIL-induced apoptosis, the significance of each rate parameter to the total model output variation can be identified by global sensitivity analysis in a matrix of 70 parameters (inputs) by 58 variables (molecular species, here the outputs) (Figure 3). The height of each bar represents the global parameter sensitivity of the corresponding species concentration with respect to changes in the reaction rate constant, or parameter. We observed that certain rate constants, such as p(1), the rate of complex formation between TRAIL and free, inactive receptor, p(29), the rate of dissociation of TRAIL and receptor, or p(57) the rate of dissociation of the activated TRAIL-receptor complex, can influence most protein concentration outputs. Therefore, some of the parameters involved in the reactions for activating the receptor complex are critical to the quantitative description of most downstream molecular species. Meanwhile other parameters have nearly zero sensitivity and thus do not affect any species concentration. Two of these parameters correspond to the reactions for dissociating the complex of the active caspase-8 and inactive caspase-3 (p(33)), and the complex of cytochrome c and the mitochondrial pores (p(48)). While these parameters will be difficult to constrain with any time series data, our analysis shows that their value should not impact model behavior. For TRAIL-induced apoptosis, the experimental data to which we aim to fit the model describes the cleavage of PARP, which marks the activation of caspase-3 and cell death. Therefore, we compared the 70 rate parameters based on their sensitivity to cleaved PARP (Figure 4, top) and observed that eight parameters had a large impact (Table 2).

**Table 2:** Comparison of results from different global sensitivity indices

	Average of local sensitivities	Sobol's first order sensitivity	Sobol's total effect sensitivity
8 dominant parameters*	k_8_, **k**_9_, k_5_, **kc**_1_, **k**_3_, k_1_, k_-_1_, k_4_	**kc**_1_, k_12_, **k**_3_, k_13_, **k**_9_, k_24_, k_10_, k_-1_	**kc**_1_, **k**_3_, k_12_, **k**_9_, k_13_, k_1_, k_10_, k_5_
CPU time	24 hours	160 hours	160 hours
Objective function^†^	3.546	8.364	7.745

* The common parameters identified by all the sensitivity indices were highlighted in bold style and the parameters by any of two different sensitivity indices were underlined.

† Global optimization (Evolutionary Strategy using Stochastic Ranking) was executed with respect to the parameter combination selected by each sensitivity index.

Importantly, there are often biologically meaningful quantities of interest for which partial derivatives cannot be defined, and these may be the outputs for which the dominant parameters need to be identified. For example, for TRAIL-induced apoptosis we can define biologically meaningful features of the dynamic behavior of cell death. One example is the delay time (t_delay_) that measures how long it takes from the time of TRAIL addition to the time at which 50% of PARP is cleaved. Another is the switching time (t_switch_), which measures the rapidity of PARP cleavage after caspase-3 (C3) activation. These features are variables that are discontinuous with respect to input parameter variation, and to determine the dominant parameters in controlling t_delay, _we therefore explored other sensitivity analysis methods to replace gradient-based sensitivity analysis.

Variance-based sensitivity methods form another category of global sensitivity analysis. In using these methods, the variance of a model output is decomposed into partial variances contributed by individual model input variations, and the sensitivity indices are derived from the ratio of the partial variance to the total variance of model output. Among the several variance-based sensitivity methods, we adopted Sobol's method [11] to analyze the TRAIL-induced apoptosis model. Sobol's method generates two kinds of sensitivity indices. One is a first-order sensitivity that measures the fractional contribution of single inputs to the variance of output, neglecting any interactions with other model inputs by maintaining these at constant values. The other, a true global sensitivity, is the total effect sensitivity, or the sum of all the sensitivities involving the model input of interest over the full range of parameters values explored. These two sensitivity indices were computed simultaneously by Monte Carlo method and the results are summarized in Figure 4. The computational cost for sensitivity analysis varies widely by method, as shown in Table 2. Sobol's method requires more computation (100,000 cases of randomly selected parameter sets) to satisfy the convergence of the Monte Carlo approximation while the average of local sensitivities method converges with 2,000 sets of parameter values.

To determine which parameters dominate the control of PARP cleavage dynamics and t_delay_, the model parameters were ranked by highest to lowest amplitude in sensitivities (Figure 4) and the eight most dominant parameters from each of the three sensitivity indices are listed in Table 2. The parameters that are commonly selected by all three methods are bolded, and those selected by two are underlined; the nomenclature of the parameters follows that of Albeck *et al*[7]. For example, k_9_, which is the forward reaction rate constant of PARP cleavage by caspase-3, is ranked within the eight dominant parameters by all three sensitivity indices. k_3 _and kc_1, _relevant to caspase-8 activation and death ligand binding to the receptor respectively, are also dominant by all three methods. Even though all the reactions in the network play a role in cell death signaling, the sets of reactions rate constants listed in Table 2 were identified as the most critical in regulating the dynamic of PARP cleavage. This prediction, that reactions relevant to caspase-8 activation are critical in regulating the delay time to death was arrived at by our computational sensitivity analysis, but, importantly, it is supported by experimental evidence: the reactions involved in caspase-8 substrate cleavage strongly influence t_delay _[19].

Once the ranking of parameters has been determined, the next question is how many parameters to target during a calibration to accurately capture network behavior. While there are no general and definitive criteria, it should be noted that estimation of too many parameters increases the number of degrees of freedom and the probability that inadequate local optima are detected. On the other hand, choosing too few parameters decreases fitting performance as well as the reliability of the optimal solution. To address this trade-off, we used the ranked parameters to determine the optimal cut-off for the calibration of the model of TRAIL-induced cell death. In Figure 4b, the 70 parameters on the x-axis were ordered by their ranking number (determined from Figure 4a). We observed that the sensitivities dropped off sharply after a few steps - ranked sensitivities generated L-shaped curves. The three different sensitivity algorithms have a common property that the parameter of 8^th ^highest sensitivity was approximately at the border between horizontal and vertical lines. This analysis suggested that for this particular model, the eight most sensitive parameters can cover much of the variation in PARP cleavage, and should be sufficient to include in model calibration.

### Parameter estimation by global optimization

**Figure 5:**
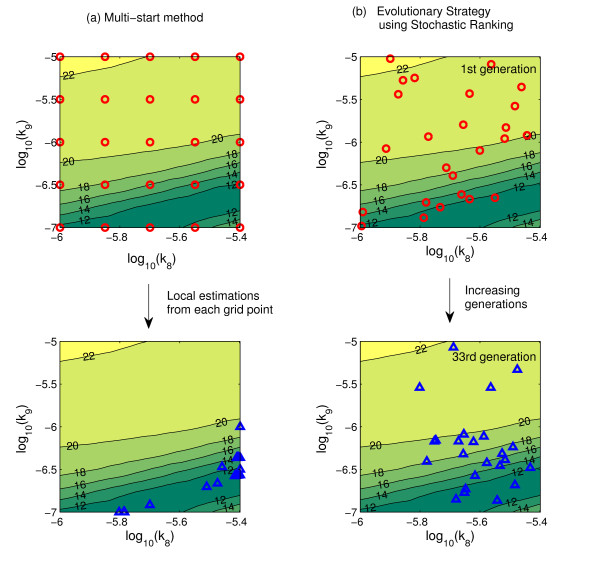
**Deterministic and stochastic sampling of 2D parameter space**. Plots show the initial and final parameter value combinations on the parameter space for k_8 _and k_9 _during global optimization process by (a) deterministic multi-start method and (b) evolutionary strategy using stochastic ranking. Starting points are indicated by red circles (top) and endpoints by blue triangles (bottom). Common contour of all sections represents the surface of the objective function.

Most models of biological processes are non-linear and thus model parameter estimations are complex problems that can have multiple solutions. To avoid potentially poor decisions made by identification of local optima, it is essential to develop a search for the global solution. Global optimization methods are roughly categorized into deterministic and stochastic approaches. A conceptual illustration of these two approaches is given in Figure 5. Here, the 2-dimensional parameter space of two rate constants (k_8 _and k_9_, in this example) was explored. As the contour of the objective function showed, there exists a valley-shaped optimum in the lower part of parameter space. It is interesting that this characteristic contour of the objective function is relevant to the discussion of dominant parameters in sensitivity analyses. The parameter k_8 _was ranked as one of the eight most influential by only one type of sensitivity analysis (average of local sensitivities), while k_9 _is ranked by all three sensitivities (Table 2). So it is expected that perturbations of k_9 _affect the model output more strongly than changes in k_8 _do. The valley-shaped contour of objective function in k_8 _vs. k_9 _parameter space indeed supports this idea, because the slope in much steeper in the k_9 _than in the k_8 _axis.

Among the various approaches for global parameter estimation, the simplest one is the deterministic multi-start method where a large number of local estimations start from different initial parameter combinations (Figure 5(a); red circles). The logarithmic space of parameters is divided uniformly in a grid and deterministic local estimation starts from every grid point, comparing the fit of nearby points. Because the entire parameter space is explored, the guarantee for finding the global optimum is high, as long as the grid samples the space sufficiently well. In Figure 5(a), parameter sets starting from initial grid points converge to the points aligned along the valley after local estimations have terminated. However the computational load increases exponentially with the number of parameters, as dimensions are added to the sampling grid. To overcome this difficulty, random sampling in a Latin hypercube of parameter space[20] or parallel computing with cluster processors could be utilized.

Stochastic methods on the other hand, can find the global solution with relatively less computational effort. These methods start with parameter values that are randomly sampled in parameter space, and, according to a set of rules, explore new solutions in the neighborhood of the initial point looking for a better solution and repeat until no further improvement of fit is found. Genetic algorithms and simulated annealing are well known examples of stochastic methods[21]. In a comparative study of various optimization methods, Stochastic Ranking Evolutionary Strategy (SRES) showed the best performance [15]. In SRES, a "population" composed of randomly selected "elements", or sets of parameter values, is generated. The elements are ranked by their fit to the data using a bubble-sort procedure[22]. Only highly ranked elements are retained as ancestors for the next generation, which are used to probabilistically produce a new population of random elements with a better fit, on average. The source code of SRES is available in the public domain[23].

**Figure 6:**
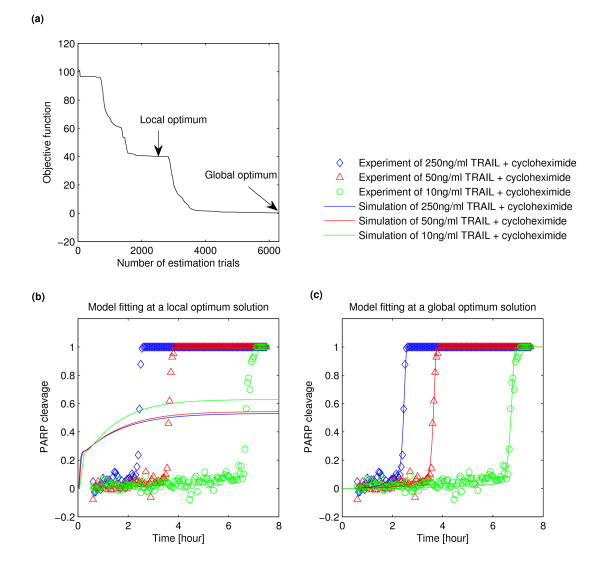
**Quantitative model calibration by multi-start method**. (a) Plot showing the multiple optimal solutions obtained by deterministic multi-start global optimization and sorted according to the magnitude of the objective function. (b, c) Plot comparing the simulated time course of PARP cleavage (lines) to single-cell data (diamond, triangle and circle markers) for cells treated with 250 ng/mL (blue), 50 ng/mL (red), or 10 ng/mL (green) TRAIL. Models simulations were derived from a local optimum (b) or a global optimum (c) of the deterministic multi-start global optimization.

For the model of TRAIL-induced cell death, we compared the performances of the deterministic multi-start method and SRES in a global optimization of the eight most dominant parameters identified by average of local sensitivities. For the multi-start method, local estimations started from the lower bound, middle value and upper bound in the range of each parameter so that the total number of cases was 6561 (= 3^8^). Out of 6561 local estimations, 6550 cases successfully detected their adjacent optimum solutions, although 11 cases failed due to their poor initial guess values. In Figure 6(a), the results of all the local estimations were sorted according to the magnitude of their objective function (see Methods for definition); every point in the curve indicates individual local optimum; the best fit had an objective function of 0.2435. The optimal parameter values are listed in Additional File 1, and fits to data are shown for a local optimum and a global optimum case (Figure 6(b) and 6(c), respectively).

It is not surprising that many local minima were detected using a multi-start method because nonlinear and complex models like cell signaling networks usually exhibits objective function surfaces with multiple local optima. The plateaus near the global optimum and around the objective function values of 40 and 100 in Figure 6(a) could be due to: 1) a wide well on the hypothetical surface of parameter space so that estimations from many nearby starting points converge to a single minimal solution, allowing us to easily arrive at the optimal solution or 2) a valley-shaped local optima on the surface of objective function. Because a wide well on the surface of parameter space is rare in network models, the most likely causes of the plateaus were valley-shaped optima. Along a valley, solutions may be distinct if they are located far apart from one another, but nevertheless fit the model in similarly well. Ideally, when constructing predictive models, this situation should be avoided by reducing valleys to more focused wells on the surface of parameter space by adding constraints to the optimization problem.

Despite its ability to find good fits to the data, the multi-start method had the critical drawback of having a heavy computational load (Table 3). As an alternative, optimization was significantly accelerated by using SRES (Table 3). For SRES, the initial population of parameter value combinations, or "elements", was generated by random selection from a uniform distribution over the 8-dimensional parameter space with the boundaries described in Methods. The population was composed of 200 individuals and, for each round, 30 individuals with best fits were defined as parents for the next generation. To decide when to terminate the optimization, we posed as a requirement a minimum of a double-digit decline in the objective function value from the first generation, and the estimation was stopped at the 33^rd ^generation, after ~2 h of computation time. Using this fast method, we compared the fits obtained by including each of the three sets of dominant parameters obtained by the different sensitivity analysis methods. We found that the set of parameters identified by the average of local sensitivities was the best, although none of the fits obtained were as good as that obtained with the deterministic multi-start method (Tables 2, 3). If, in the case of the parameters identified with the average of local sensitivities, we allowed the evolution to proceed further, the fit did improve very slowly while the CPU usage time increased significantly (Table 3). To obtain a goodness-of-fit equivalent to that achieved with the deterministic multi-start method, we implemented a previously described hybrid method[24]. Using this method, the optimization was carried out in two sequential phases: first, a local solution in the vicinity of the global optimum was rapidly reached by the SRES method and, second, the solution was refined by a fast local estimation method until a pre-defined tolerance was satisfied (see Methods). As Table 3 shows, this hybrid method could fit the model in much less computation time than the deterministic multi-start method, with an objective function as good as that obtained with the laborious multi-start method. Generally, the choice of optimization methods is dependent on not only model type but also on resource availability or approximation tolerance. Each method may have different performance for different models. With respect to the model in this study, the combination of SRES and local estimation performed the most efficient survey of the parameter space in a global optimization results. This efficiency was due to its combination of rapid stochastic surveying of the whole space and deterministic searching within local regions.

**Table 3:** Comparison of estimation performance by different optimization algorithms

	Local optimization	Multi-start of local optimization	**SRES**^†^**(G^‡ ^= 33)**	SRES (G = 300)	SRES (G = 33) & local optimization
Number of surveyed parameter combinations	1	6561	6600	60000	6600

CPU time	~1 minute	2600 hours	2 hours	18 hours	2 hours

Objective function	45.04	0.2447	3.546	1.739	0.2679

^† ^SRES, Evolutionary Strategy using Stochastic Ranking;^‡ ^G, generation number of evolution.

### Influence of dominant parameter choice on optimization

**Figure 7:**
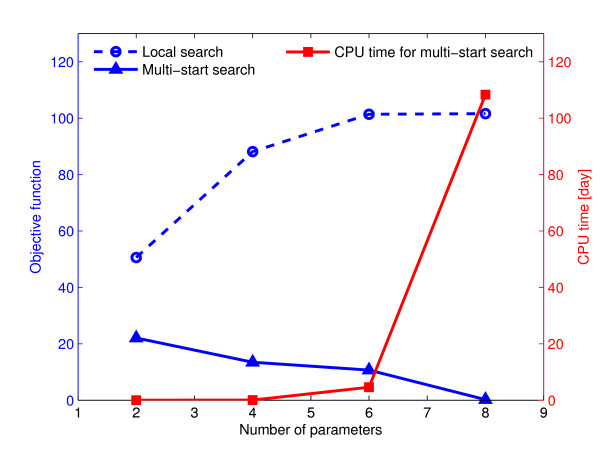
**Effect of number of parameters on model calibration performance**. Effect of varying the number of parameters included in model calibration on model fit, considering local or multi-start deterministic search and CPU usage time for global multi-start optimization.

To validate our choice of eight dominant parameters to estimate, we examined goodness-of-fit and computational cost while varying the number of parameters to be estimated for two deterministic optimization methods, where the number of parameters optimized has the greatest impact on computation time. In Figure 7, we show CPU time for the multi-start search and optimal objective function values as a function of the number of parameters estimated, for both the local search and the multi-start search. The fit to the data at the global optimum solution detected by multi-start search improved with increasing number of parameters, reaching a plateau at eight parameters, while computational cost increased exponentially. Importantly, the performance of the local search deteriorated significantly when the number of parameters increased. This is because when the local search starts from a poor initial guess, the chance of arriving at local optimum solutions with poor fitting performance increases, and with a larger parameter space to sample, the local search is more likely to start from a poor initial guess. This result shows how important it is to apply a global, or hybrid, optimization algorithm to obtain the best fits (Table 1), or to adequately limit the search space when using a local search. Overall, the good performance (using a multi-start search) and affordable computational cost lead us to conclude that choosing the eight parameters identified as dominant in global sensitivity analysis for quantitative model fitting was indeed an appropriate compromise. Fitting more than eight parameters for the TRAIL model optimization would yield only little improvement in fit, at much greater computational cost. The number of dominant parameters in a particular model would certainly be dependent on its size and complexity, but the sensitivity analysis-based method described above allows their identification.

**Figure 8:**
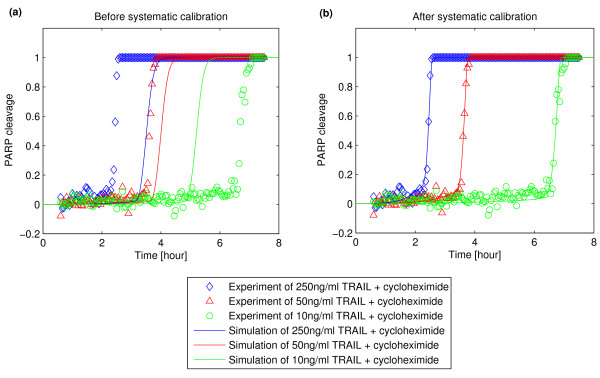
**Improvement of model fitting by systematic calibration process**. (a) Model fitting with the basal rate constants which were manually calibrated [7]. (b) Model fitting with the estimated rate constants by the systematic calibration process.

Finally, Figure 8 shows how much the model improved using our method relative to the manual calibration used in the original study [7]. It is noteworthy that adjustment of a few important parameters could substantially improve agreement between model output and experimental data. The procedure to identify those important parameters and estimate them is straightforward by systematic methodology compared to manual calibration which is inevitably labor-intensive and time-consuming with less guarantee of successful model fitting.

## Conclusion

In this report, we proposed a framework for efficiently calibrating computational models of biological systems, and applied it to a model of TRAIL-induced apoptosis while comparing several sensitivity analysis methods and model optimization algorithms. Importantly, we showed how sensitivity analysis can be used to rapidly test whether the model structure adequately allows qualitative matching to the behavior of the biological system. This step implements a minimal qualification, focusing the initial search on the qualitative performance of the proto-model. Within our framework, this validation step is required before proceeding to quantitative optimization of the model, ensuring that computationally costly optimization algorithms are used effectively. Furthermore, we showed how global sensitivity analysis methods can be used to identify the parameters that dominantly regulate the dynamics of the output of interest. With the application of Sobol's algorithms, we were also able to identify parameters that control the TRAIL-induced delay time to cell death (t_delay_), a biologically relevant quantity that is not a state variable of the model. Undoubtedly, this type of sensitivity analysis will prove useful within our outlined framework for other models as well, for example in models of oscillatory systems where, in certain cases, the period of the oscillations is more meaningful than their amplitude. Finally, while comparing different model calibration algorithms, we showed that global sensitivity analysis could successfully identify the parameters to include in quantitative optimization, allowing great computational savings by constraining the search to the important model dimensions. In the future, we foresee that the predictive quality of models would be further improved by repeating this cycle of model validation, identification of dominant parameters and optimization with different model outputs that are controlled by other parameters, allowing the determination of more and more parameter values.

## Methods

### Mathematical model and experimental data

The serial methodology was applied to fit a recently described mathematical model of TRAIL-induced cell death signaling [7]. This model is composed of ordinary differential equations based on mass action kinetics. Although most ODE models assume simply that the inside of cell is a mixed soup and do not include spatial information, the original model describes reactions and transport of molecular species in two compartments; cytoplasm and mitochondria. The authors verified that sudden activation of effector caspase after a long delay is related to permeabilization of the mitochondrial membrane and relocalization of certain proteins. To evaluate our methodology, we carried out model calibration using the original ODE model and the experimental data. The live-cell imaging data were obtained by microscopy monitoring of a population of HeLa cells treated with 10 ng/ml, 50 ng/ml, or 250 ng/ml of TRAIL and 2.5 μg/ml cycloheximide [7]. Although the cells were isogenic, the delay period until sudden death (t_delay_) varied from cell to cell due to inherent fluctuations in cell state [19]. Figure 2B in [7] actually shows 5 examples of single-cell dynamic data for each condition; these examples were chosen after monitoring well over 100 cells in each condition. However we focus here on deterministic modeling at the single-cell level. Thus, for each condition we chose a single representative cell whose t_delay _is the median in the population of more than 100 cells. The cleavage of effector caspase reporter protein (EC-RP) was quantified at 3-min intervals, and used for fitting the model output corresponding to cleavage of PARP, the effector caspase substrate. In addition, once the rapid cleavage of EC-RP is complete, then the output signal cannot be accurately measured by microscopy - it becomes extremely noisy as the cells detaches from the surface and moves out of the focal plane, and thus poorly reports cellular activity. Therefore we neglected the fluctuations in the experimental data after cleavage of PARP reaches a value of 1. Instead, we fit the model to a plateau with a value of one.

We used the weighted least squares method for parameter estimation. The objective function to minimize is

where *p *is the set of parameters, *N*_exp _is the number of experiments, *w*_*i *_is the weight associated with the measurement of the i^th ^experiment, , and *y*_*i*_(*p*) is the corresponding value computed from the model. The weight may be given differently depending on reliability of specific experimental measurement. If there are uncertain or less confident data points, those should take less part in evaluating the objective function by using smaller weight than other data points. Since we took all the data with equal importance, the weights were all set equal to 1 in this case. The estimation calculation was stopped if the normalized difference of objective function values between two successive iterations was less than 1E-6.

### Parameter space

The plausible range for uncertain rate constant parameter values was set around a nominal value of the corresponding parameter. For most parameters, the upper and lower bounds were set as 100 and 1/100 times the nominal value, respectively. For several parameters whose nominal values are considered to be relatively certain by previous experiences we set narrower ranges to minimize the effect of uncertain parameter values in the global sensitivity analysis. For instance, forward, backward and catalytic rate constants relevant to caspase-3 cleavage by caspage-8 (k_5_, k_–5_, kc_5_), caspase-6 cleavage by caspage-3 (k_6_, k_–__6_, kc_6_), PARP cleavage by caspase-3 (k_9_, k_–9_, kc_9_), Bid activation by cleaved caspase-8 (k_10_, k_–__10_, kc_10_), Bcl2 reacting with activated Bax in the mitochontrial compartment (k_14_, k_–14_), Bax_2 _reacting with Bcl2 (k_16_, k_–16_), Bax4 reacting with Bcl2 (k_18_, k_–__18_), Cytochrome c and Smac release from mitochondria (k_20_, k_–__20_, kc_20_, k_21_, k_–21_, kc_21_) had a range set to between 10 and 1/10 times of their nominal value. The rate constants regarding ubiquitination of cleaved caspase-3 by XIAP (k_8_, _k–__8_, kc_8_) and XIAP reacting with the apoptosome and Smac (k_27_, k_–__27_, k_28_, k_–28_) have an even narrower range between 2 and 1/2 times the nominal value. The nomimal values were either obtained from the literature or set by trial and error to allow the model to reproduced experimental data, as previously described [7].

### Computations

All computations were performed using JACOBIAN^® ^4.0, a dynamic modeling software provided by Numerica Technology, LLC. The local estimation was executed by the built-in JACOBIAN^® ^function of Limited memory Broyden-Fletcher-Goldfarb-Shanno (L-BFGS) estimation solver and a weighted least square objective function. The High-Performance Computing facility at Harvard Medical School was utilized for intensive computations. The repetitive jobs of the multi-start estimation as well as Sobol's sensitivity analysis were parallelized and distributed to over 200 computing nodes (AMD dual core processors). For comparisons between different algorithms, the CPU usage time of each node was summed as if a single computing machine was utilized.

## Authors' contributions

KAK performed the simulations and SLS, SG, JGA and JMB contributed to analysis and interpretation of data. KAK wrote the draft and SLS, SG, and JGA revised the manuscript critically. PKS and DHK conceived of the study and participated in its design and coordination. All authors read and approved the final manuscript.

## Supplementary Material

Additional file 1 **The dominant parameters and the estimated results**. This pdf file details the dominant parameters concerning their related reactions, parameter ranges and estimated values by different optimization methods. Click here for file
