# Supplementary material for: Systematic calibration of a cell signaling network model
Source: BMC Bioinformatics. 2010 Apr 23;11:202. doi: 10.1186/1471-2105-11-202 (PMC2880028; doi:10.1186/1471-2105-11-202)
Supplement: Additional file 1 — The dominant parameters and the estimated results. This pdf file details the dominant parameters concerning their related reactions, parameter ranges and estimated values by different optimization methods. [file 1471-2105-11-202-S1.PDF]

The dominant parameters and the estimation results by different estimation methods

Parameter	Reaction	Estimation range	Local optimum by multi-start	Global optimum by multi-start	SRES[†] ($G^{‡}$=33)	SRES (G=300)	SRES (G=33) & local optimization
k_8	C3* + XIAP -> C3*:XIAP	$1\times10^{-6} \sim 4\times10^{-6}$	3.24×10^{-6}	4.00×10^{-6}	3.55×10^{-6}	3.56×10^{-6}	3.59×10^{-6}
k_9	C3* + PARP -> C3*:PARP	$1\times10^{-7} \sim 1\times10^{-5}$	1.00×10^{-5}	9.81×10^{-6}	1.70×10^{-6}	1.02×10^{-6}	1.68×10^{-6}
k_5	C8* + C3 -> C8*:C3	$1\times10^{-8} \sim 1\times10^{-6}$	7.05×10^{-7}	5.63×10^{-7}	2.53×10^{-7}	3.11×10^{-7}	2.53×10^{-7}
kc_1	L:R -> R*	$1\times10^{-7} \sim 1\times10^{-3}$	1.00×10^{-3}	1.00×10^{-3}	3.75×10^{-4}	4.81×10^{-7}	3.71×10^{-4}
k_3	R* + C8 -> R*:C8	$1\times10^{-8} \sim 1\times10^{-4}$	1.00×10^{-4}	1.46×10^{-8}	1.50×10^{-6}	3.16×10^{-6}	1.49×10^{-6}
k_1	L + R -> L:R	$4\times10^{-9} \sim 4\times10^{-5}$	4.00×10^{-5}	7.16×10^{-7}	1.60×10^{-8}	5.42×10^{-6}	3.83×10^{-8}
k_{-1}	L:R -> L + R	$1\times10^{-5} \sim 1\times10^{-1}$	1.00×10^{-1}	1.00×10^{-3}	7.02×10^{-2}	2.83×10^{-4}	1.45×10^{-1}
k_4	C8* + Bar -> C8*:Bar	$1\times10^{-8} \sim 1\times10^{-4}$	1.00×10^{-6}	3.89×10^{-5}	5.52×10^{-5}	8.43×10^{-5}	1.14×10^{-4}

[†] SRES, Evolutionary Strategy using Stochastic Ranking; [‡] G, generation number of evolution.
